# Comparative Genomic Analysis Uncovers the Evolutionary Basis of Siliceous Cell Wall Formation Across Diverse Lineages

**DOI:** 10.3390/biology15141127

**Published:** 2026-07-10

**Authors:** Limin Jia, Liangwei Li, Yaolei Zhang, Jiahao Wang, Zengbao Yuan, Guangyi Fan, Chengcheng Shi, Man Zhang

**Affiliations:** 1 College of Life Sciences, University of Chinese Academy of Sciences, Beijing 100049, China; jialimin_1999@163.com; 2 BGI-Qingdao, Qingdao 266555, China; liliangwei@genomics.cn (L.L.); zhangyaolei@genomics.cn (Y.Z.); wangjiahao@genomics.cn (J.W.); fanguangyi@genomics.cn (G.F.); 3 College of Fisheries, Henan Normal University, Xinxiang 453007, China; 4 BGI-Shenzhen, Shenzhen 518083, China; 5 College of Fisheries, Southwest University, Chongqing 400715, China; yuanzengbao1998@163.com

**Keywords:** comparative genomics, siliceous cell wall, biomineralization, silicon transporter, horizontal gene transfer, diatom, evolution

## Abstract

In this study, we constructed a comparative genomic framework encompassing 57 genome sequences from four key taxonomic groups—Bacillariophyta, Parmales, choanoflagellates, and *Bacillus*—all of which possess either siliceous cell walls or silicon-transporting vesicle structures. By comparing these genomes with those of non-silicified species, including Chlorophyta, Streptophytes, Rhodophyta, and Dinoflagellates, we systematically analyzed the evolutionary distribution patterns of genes involved in silicon transport, silicification, and related processes across eukaryotes. Through orthogroup clustering and phylogenetic analysis, we identified 75 orthogroups universally conserved across all 57 species (including representing siliceous and non-siliceous groups), and an additional 105 orthogroups consistently present across the four silica-bearing lineages (Bacillariophyta, Parmales, choanoflagellates, and *Bacillus*), which were predominantly enriched in fundamental metabolic pathways. Furthermore, by integrating 120 known siliceous cell wall-related protein sequences, we identified three orthogroups broadly distributed across the four major lineages, suggesting an ancient evolutionary origin of certain silicon-related genetic components. Our findings provide genomic insights into the evolutionary trajectory of siliceous cell wall-associated genes and offer a valuable resource for future studies on biomineralization in eukaryotes.

## Simple Summary

Siliceous cell walls are key biomineralization structures in eukaryotes, particularly in diatoms, but their evolutionary origins remain poorly understood. In this study, we identified 75 orthogroups universally conserved across 57 species (representing eight taxonomic groups), and an additional 105 orthogroups consistently present across four silica-bearing lineages (Bacillariophyta, Parmales, choanoflagellates, and *Bacillus*), mainly associated with fundamental metabolism. Among 120 known silicification-related proteins, several ancient silicon-related genes were found to be shared across all silicifying lineages, indicating an ancient origin of silicon-related genetic machinery. Silicon transporters and structural proteins exhibited distinct evolutionary patterns, including lineage-specific duplications and horizontal gene transfers. Our results provide new insights into the evolutionary assembly of siliceous cell wall genetic toolkits in eukaryotes.

## 1. Introduction

Siliceous cell walls represent one of the most remarkable biomineralization structures in eukaryotes, with diatoms being the most prominent example. These photosynthetic microalgae produce intricately patterned silica-based cell walls that not only provide mechanical protection but also play a central role in global biogeochemical cycles, accounting for an estimated 20% of global primary productivity and serving as a major sink for marine silicon [1,2]. The evolutionary origin of this sophisticated biomineralization capability has long fascinated biologists, yet the genetic underpinnings of siliceous cell wall formation remain incompletely understood.

The ability to deposit silica is not unique to diatoms. Several other eukaryotic lineages, including choanoflagellates—the closest living relatives of animals—produce siliceous scales or basket-like lorica structures [3]. Parmales, a group of heterotrophic flagellates that occupy a key phylogenetic position near the root of the Opisthokonta, have also been reported to possess silicon-transporting vesicles, although they do not form structured siliceous cell walls [4]. Interestingly, even some bacteria, such as *Bacillus* species, are capable of silica deposition under specific environmental conditions [5]. The presence of silica-related traits across such phylogenetically distant lineages raises fundamental questions about the evolutionary history of the underlying genetic machinery: Are the genes involved in silicon transport and deposition shared among these diverse organisms, or have they arisen independently through convergent evolution?

Comparative genomics offers a powerful approach to address these questions by tracing the distribution and evolutionary trajectories of candidate genes across diverse taxonomic groups [6]. Previous studies have identified a number of genes involved in silicon metabolism in diatoms, including silicon transporters (SITs) [1], silaffins [2], and long-chain polyamine synthesis enzymes [7]. However, most of these investigations have focused on individual lineages, particularly diatoms, and have not systematically examined the presence or absence of these genes in closely related non-silicified taxa. A notable exception is the recent discovery that SIT homologs exist in choanoflagellates and other non-silicified eukaryotes [8,9], suggesting that some components of the silicon-related genetic toolkit may be more ancient than previously appreciated. Nevertheless, a comprehensive comparative analysis that simultaneously incorporates key silicified and non-silicified lineages across the eukaryotic tree remains lacking.

Diatoms, as one of the most successful eukaryotic groups, have evolved diverse adaptive strategies, including silicification, which has contributed to their ecological dominance [10]. The genetic basis of silicification has been partially unraveled through transcriptomic and proteomic studies, revealing a complex network of genes involved in silica transport, precipitation, and cell wall assembly [11]. However, the evolutionary conservation and diversification of these genes across eukaryotes remain poorly characterized. Horizontal gene transfer (HGT) has been increasingly recognized as a significant driver of eukaryotic genome evolution, facilitating the acquisition of novel traits, including biomineralization capabilities [12,13]. The potential role of HGT in the evolution of silicon-related genes across distantly related lineages, however, remains largely unexplored.

To address this gap, we assembled a dataset of 57 genomes representing four key groups with documented silica-related traits—diatoms, Parmales, choanoflagellates, and *Bacillus*—alongside non-silicified reference species from green algae, streptophytes, red algae, and dinoflagellates. By integrating orthogroup clustering with targeted analysis of known siliceous cell wall-associated genes, we aimed to achieve two main objectives. First, we sought to identify core orthogroups that are universally conserved across all sampled species, thereby defining the shared genetic foundation upon which silica-related traits may have been built. Second, we systematically examined the distribution patterns of known siliceous cell wall-related genes across these diverse lineages, with a particular focus on identifying genes that are broadly distributed beyond silicified taxa. Our results provide a genome-wide perspective on the evolutionary architecture of silicon-related genetic components and offer new insights into the deep evolutionary history of biomineralization in eukaryotes.

## 2. Materials and Methods

### 2.1. Genome Data Collection

Genome sequences and associated annotation files were collected from three public repositories: the National Center for Biotechnology Information (NCBI; https://www.ncbi.nlm.nih.gov/, Bethesda, MD, USA) (accessed on 15 January 2025), the China National GeneBank (CNGB; https://db.cngb.org/, Shenzhen, China) (accessed on 15 January 2025), and the Joint Genome Institute (JGI; https://genome.jgi.doe.gov/portal/, Walnut Creek, CA, USA) (accessed on 15 January 2025). To ensure data quality for subsequent comparative analyses [1,2,8], genomes derived from environmental sequencing projects (e.g., metagenome-assembled genomes or single-cell amplified genomes) were excluded, as these assemblies often contain fragmentation issues and incomplete gene sets that could confound orthogroup inference [14].

A total of 57 genomes were selected to represent four key lineages with documented silica-related traits or evolutionary relevance to siliceous cell wall formation. These included: (i) diatoms (Bacillariophyta, 25 species); (ii) Parmales (8 species); (iii) choanoflagellates (Choanoflagellata, 2 species); (iv) *Bacillus* (10 species). Siliceous sponges were not included in this study because currently available sponge genome assemblies are fragmented and incompletely annotated, which could introduce biases in orthogroup clustering and gene presence/absence calls. In addition, our primary focus was to compare lineages with high-quality reference genomes. Non-silicified reference species were included from green algae (Chlorophyta, 4 species), streptophytes (Streptophyta, 2 species), red algae (Rhodophyta, 3 species), and alveolates (Alveolata, 3 species) to serve as comparative outgroups. A complete list of the 57 species, including accession numbers and assembly statistics, is provided in Table 1; Supplementary Table S1.

### 2.2. Genomic Annotation and Quality Control

The genomic datasets for all 57 species underwent comprehensive annotation following a standardized pipeline [15,16]. Repeat sequences were identified using RepeatMasker v4.0.6 [17] and RepeatModeler v1.0.8 [18]. Gene structural prediction employed a hybrid approach that combined ab initio prediction using Augustus v3.1.0 [19] and GeneMark v4 [20]) with homology-based prediction using Genewise v2.4.1 Integration and optimization of the predictions were performed using EvidenceModeler to generate the final gene structure models. For species with publicly available high-quality annotations from the source databases (NCBI, CNGB, or JGI), we retained the original annotations after verifying consistency with our quality assessment criteria. Annotation completeness was evaluated by examining the proportion of conserved single-copy orthologs using BUSCO v5 [21]), with the lineage-specific database selected for each taxonomic group according to the closest available reference (Supplementary Table S1).

### 2.3. Orthogroup Clustering Across Multiple Lineages

**Figure 1:**
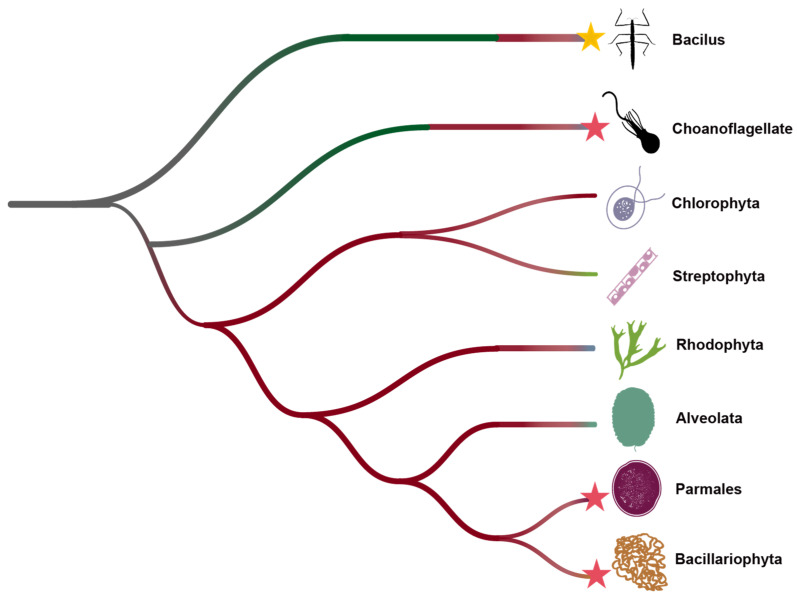
Phylogenetic relationships and genomic information of the 57 species included in this study. Branch colors indicate taxonomic groups: blue, Bacillariophyta; orange, *Bacillus*; purple, Parmales; pink, Choanoflagellata; green, Chlorophyta; light green, Streptophyta; red, Rhodophyta; brown, Alveolata. Red stars indicate lineages with documented siliceous cell walls (Bacillariophyta, Parmales, and Choanoflagellata); yellow stars indicate *Bacillus* species, which possess silica precipitation capabilities but do not form structured siliceous cell walls.

**Figure 2:**
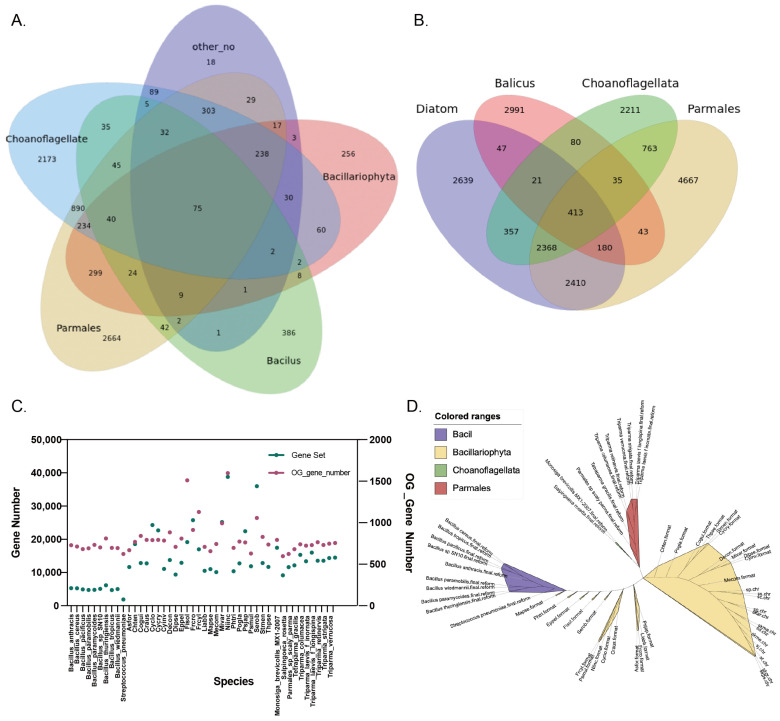
Core orthologous gene families shared by all 57 species. (**A**) Venn diagram of orthogroups shared between silicified (35 species) and non-silicified (22 species) lineages. (**B**) Venn diagram of orthogroup distribution among the four silica-bearing lineages. (**C**) Copy number distribution of 413 orthogroups present in ≥50% of species within each silica-bearing lineage. Red circles: per-species gene counts; green circles: total gene counts per orthogroup. (**D**) Maximum likelihood phylogenetic tree of the four silica-bearing lineages constructed from 105 universally conserved single-copy orthogroups. Bootstrap support values (≥70%) are shown at nodes.

To investigate the conservation and distribution of gene families across diverse evolutionary lineages, we performed orthogroup clustering using OrthoFinder v2.5.4 [22] on the complete protein sequences of all 57 genomes. The analysis was conducted with default parameters unless otherwise specified: the inflation value for MCL clustering was set to 1.5, and the sequence similarity search was performed using DIAMOND v2.0.15 [23] with an E-value threshold of 1 ×10^−5^. The resulting orthogroup assignments were compiled into a presence/absence matrix across all species, which was subsequently visualized as a Venn diagram to illustrate the distribution of orthogroups shared among the eight taxonomic groups (Figure 1 and Figure 2A). This analysis revealed 75 orthogroups that were conserved across all eight lineages, a relatively low number likely attributable to the deep phylogenetic distances among these groups.

Given our focus on lineages with documented silica-related traits, we performed a second orthogroup clustering analysis restricted to the four key groups that possess either siliceous cell walls or silicon-transporting vesicle structures: diatoms (Bacillariophyta), choanoflagellates (Choanoflagellata), parmales (Parmales), and *Bacillus* species. OrthoFinder v2.5.4 was applied to the protein sequences of these four groups using the same parameter settings described above. The resulting orthogroup presence/absence matrix was visualized as a Venn diagram to capture the distribution of gene families among these four lineages.

We acknowledge that the inclusion of both bacterial and eukaryotic genomes in the same orthogroup and phylogenetic framework requires careful handling, given the deep evolutionary distances, divergent genome structures, and differences in annotation quality between these lineages. To minimize potential artifacts, we implemented several additional measures. First, all genomes were assessed using BUSCO with lineage-specific databases; only genomes with completeness ≥70% (for eukaryotes) or ≥95% (for bacteria) were included (see Supplementary Table S1 for BUSCO scores). This filtering step ensured that poor-quality assemblies or annotations did not compromise orthogroup inference. Second, the ≥50% species presence threshold was chosen to tolerate annotation gaps and incomplete assemblies—which are more common in eukaryotic genomes—while maintaining sufficient stringency to exclude lineage-specific or species-specific gene families that could arise from annotation errors. Third, OrthoFinder was run with an MCL inflation value of 1.5, balancing sensitivity against over-splitting of orthogroups, and DIAMOND was used with sensitive mode to improve remote homology detection across deep phylogenetic distances, yielding a set of 413 orthogroups. Within this set, we examined the distribution of gene copy numbers per species, visualized as a line plot with red lines representing the five silicified and silica-vesicle-bearing groups (diatoms, choanoflagellates, and Parmales) and green lines representing *Bacillus* species. Copy numbers were generally consistent among the eukaryotic groups, whereas *Bacillus* species exhibited a notably lower total gene count.

Further filtering of the 413 orthogroups identified a subset of 105 orthogroups that were present in all species across the four silica-related lineages, representing a core set of universally conserved gene families among these groups. Protein sequences from these 105 orthogroups were extracted and aligned using MAFFT v7.475 [24] with the L-INS-i strategy. Phylogenetic trees were constructed for each orthogroup using IQ-TREE v2.2.0 [25]. The resulting phylogenetic trees were used to reconstruct a robust species tree (Figure 1) and to confirm the single-copy status of these core orthogroups across all 57 species. No direct functional link to silicon metabolism is claimed for these core orthogroups; rather, they represent the essential cellular machinery common to all sampled lineages.” It is important to note that these two numbers reflect different inclusion criteria: the 75 orthogroups are present in all 57 species across all eight lineages, whereas the 105 orthogroups are present in all species within the four silica-bearing lineages but may be absent in non-silicified outgroups.

### 2.4. Phylogeny of Silicon-Related Gene Families

Homologous sequences were identified using DIAMOND v0.9.21.122 [23] BLASTp search against the NCBI NR database, and the top 1,000 hits were retained. To ensure balanced taxonomic representation, filtering was applied to retain no more than 12 sequences per phylum, no more than 5 sequences per genus, and a single sequence per species. The filtered sequences were aligned using MAFFT v7.299 [24] and trimmed with trimAl v1.4 [26]. A maximum likelihood tree was constructed using IQ-TREE v2.1.4 [25]. The tree was midpoint-rooted using the ape and phangorn R packages and visualized with iTOL v6 [27].

For homolog identification of the 120 known siliceous cell wall-associated proteins across the 57 genomes, DIAMOND BLASTp searches were performed with an E-value threshold of 1 ×10^−5^ against the proteomes of all 57 genomes. Homologs were retained when sequence identity was ≥30% and query coverage was ≥50%. Reciprocal BLAST was not used because our primary aim was to detect presence/absence across diverse lineages rather than to establish strict one-to-one orthology, which is particularly challenging across deep phylogenetic distances. Functional assignments were confirmed using Pfam domain annotations via eggNOG-mapper where available. For NR database searches (Figure 4A), the same thresholds were applied, with additional taxonomic filtering to ensure balanced representation across phyla.

Among the 120 known siliceous cell wall-associated proteins, we performed detailed phylogenetic analysis specifically on two categories: silicon transporters (SITs, 57 sequences) and structural proteins (32 sequences). These two categories were selected because they are the best-annotated, longest in sequence length, and most directly relevant to silicon uptake and cell wall formation. The remaining categories (e.g., secretion-related proteins, polyamine synthesis enzymes) were too short or insufficiently conserved across deep evolutionary distances to yield reliable alignments and well-resolved trees; they are therefore presented only in the presence/absence analysis (Figure 4B).

### 2.5. Functional Annotation Analysis

Functional annotations for all protein sequences were obtained using eggNOG-mapper v2.1.9 [28]. GO functional annotations were performed using topGO v2.46.0 [29] with the weight01 algorithm, considering biological process, molecular function, and cellular component categories separately. KEGG pathway functional annotations were conducted using clusterProfiler v4.2.2 [29]. For both analyses, result was assessed using Fisher’s exact test or hypergeometric testing, with FDR-adjusted *p*-value < 0.05 as the significance threshold. Focused functional annotations were performed on the 105 core orthogroups to elucidate their functional roles. For the three broadly distributed orthogroups, we present functional annotation (based on eggNOG-mapper and KO assignments) rather than statistical enrichment, as the small gene set size precludes meaningful enrichment testing.

## 3. Results and Discussion

### 3.1. Phylogenetic Relationships and Genomic Information of the 57 Species Genome Included in This Study

To establish a robust comparative framework for investigating the evolutionary distribution of siliceous cell wall-associated genes, we assembled a dataset comprising 57 genomes representing eight major taxonomic groups (Figure 1; Table 1). These included the four key lineages with documented silica-related traits: Bacillariophyta (25 species), Parmales (8 species), Choanoflagellata (2 species), and *Bacillus* (10 species). Non-silicified reference species were also included as outgroups, comprising Chlorophyta (4 species), Streptophyta (2 species), Rhodophyta (3 species), and Alveolata (3 species). This sampling strategy allowed us to compare genome content across lineages that differ in their capacity for silica biomineralization while controlling for phylogenetic distance.

A maximum likelihood phylogenetic tree was constructed based on a concatenated alignment of 105 single-copy orthogroups that were universally conserved across all 57 species (Figure 2A). The resulting tree topology was consistent with established eukaryotic phylogeny, with clear separation of the major lineages [30,31,32]. Within the Bacillariophyta clade, the centric diatoms (Coscinodiscophyceae) showed a closer phylogenetic affinity to choanoflagellates and Parmales, whereas the remaining Bacillariophyta lineages—including the pennate diatoms (Bacillariophyceae)—exhibited a closer relationship to *Bacillus* species, while *Bacillus* species formed a distinct outgroup branch, reflecting their distant phylogenetic position as bacteria.

### 3.2. Core Orthologous Gene Families Shared by All 57 Species and Their Functional Characteristics

Orthogroup clustering identified 75 orthogroups that were universally conserved across all 57 species (Figure 2A). Separately, 105 orthogroups were found to be present in all species across the four silica-bearing lineages (Bacillariophyta, Parmales, choanoflagellates, and *Bacillus*; Figure 2C). The latter set was used for phylogenetic reconstruction (Figure 2D). To identify the genetic foundation common to all species in our dataset, we performed orthogroup clustering using OrthoFinder on the protein sequences of all 57 genomes. We first emphasize that the core orthogroups described below are not claimed to be directly involved in silicon metabolism. Instead, they serve two specific purposes in this study. First, they provide a set of universally conserved single-copy markers for reconstructing a robust phylogenetic tree of all 57 species (Figure 1), which establishes the evolutionary framework for subsequent analyses. Second, they demonstrate that all sampled lineages—regardless of their silicification capacity—share a conserved set of core metabolic genes, providing essential background context for understanding how lineage-specific expansions of silicon-related genes occurred on top of this conserved machinery [33,34]. Orthogroup clustering identified 75 orthogroups that were universally conserved across all 57 species, representing the core genetic components shared across this phylogenetically diverse dataset. In addition, 40 orthogroups were found to be specifically conserved among the four silica-bearing lineages but absent or highly diverged in non-silicified outgroups. We refer to these as ‘lineage-restricted orthogroups’ rather than assuming a direct role in silicification. Functional annotation of these 40 lineage-restricted orthogroups revealed genes involved in general cell wall organization and membrane lipid synthesis, including a glycosyltransferase (EXT1, K02366) and a sulfoquinovosyltransferase (K06119) [35].

A total of 413 orthogroups were identified as being present in at least 50% of the species within each of the four silica-bearing lineages—Bacillariophyta, Parmales, choanoflagellates, and *Bacillus* (Figure 2B). Analysis of gene copy numbers within these 413 orthogroups revealed a consistent pattern across eukaryotic species (Bacillariophyta, Parmales, and choanoflagellates), with most orthogroups maintained as single-copy or low-copy genes (red line in Figure 2C). This uniformity suggests that these broadly distributed orthogroups are under strong evolutionary constraint, limiting gene duplication and retention events [36,37,38].

In contrast, *Bacillus* species exhibited substantially lower total gene counts within these orthogroups (green bubble in Figure 2C). This reduction is likely attributable to the compact genome architecture of bacteria, which typically undergo genome streamlining to eliminate redundant or non-essential genetic elements. The lower copy numbers in *Bacillus* may also reflect differences in cellular complexity and regulatory requirements between prokaryotes and eukaryotes. Among the 413 broadly distributed orthogroups, 105 were found to be universally present in all species across the four lineages, representing a core set of essential genes that have been conserved through deep evolutionary timescales. This analysis revealed a total of 105 orthogroups that were present in every species across the four silica-bearing lineages (Figure 2C). These core orthogroups represent the minimal set of gene families that have been maintained throughout the evolutionary divergence of these lineages, spanning both eukaryotes and bacteria.

Examination of the presence/absence patterns of these 105 orthogroups revealed that the vast majority were present as single-copy genes in most species (Figure 2D). Only a small subset exhibited lineage-specific duplications, with certain orthogroups showing increased copy numbers in diatom genomes compared to other groups. The conservation of these orthogroups across such phylogenetically distant lineages—including bacteria, excavates, and multiple eukaryotic supergroups—suggests that they fulfill essential cellular functions that are required for fundamental biological processes.

**Figure 3:**
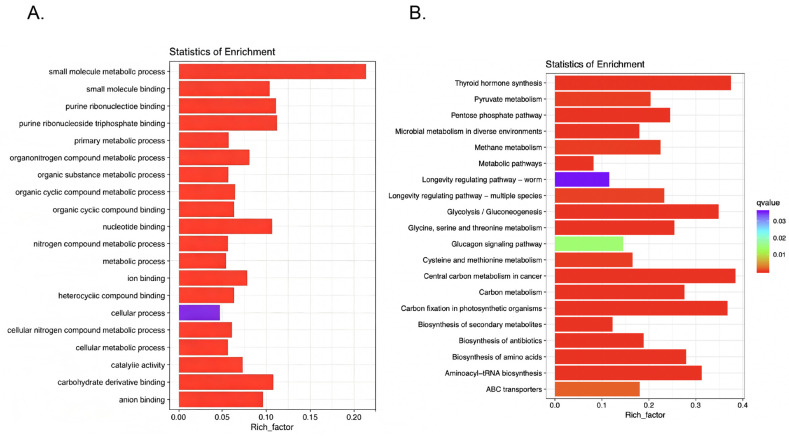
(**A**) GO functional annotations of 302 core orthogroup genes in Phaeodactylum tricornutum. (**B**) KEGG pathway functional annotations of the same 302 genes. Bar color indicates statistical significance (*q*-value).

To further elucidate the functional roles of the 105 core orthogroups in a representative diatom species, we mapped these orthogroups to the genome of Phaeodactylum tricornutum. A total of 302 genes in *P. tricornutum* were assigned to the 105 core orthogroups and were subjected to Gene Ontology (GO) and Kyoto Encyclopedia of Genes and Genomes (KEGG) functional annotations (Figure 3A,B).

KEGG pathway functional annotations further supported these functional assignments, with core genes showing significant enrichment in multiple metabolic pathways. Notably, “pyruvate metabolism” (ko00620) was among the most significantly enriched pathways, highlighting the central role of these core orthogroups in energy metabolism. This enrichment underscores the critical function of these core genes in maintaining cellular energy homeostasis across diverse lineages.

### 3.3. Distribution Patterns of Known Siliceous Cell Wall-Associated Genes Across Four Major Lineages

To investigate the evolutionary distribution of genes involved in silica biomineralization, we assembled a curated set of 120 known siliceous cell wall-associated protein sequences derived from previous studies on diatom model species (Supplementary Table S1) [39,40]. These sequences were classified into five functional categories based on their known or predicted roles in silicon metabolism: (i) silicon transporters (SITs), which mediate the uptake of silicic acid from the environment; (ii) silaffins and other structural proteins (sillaffins, cingulins, and silacidins), which constitute the organic matrix of the siliceous cell wall; (iii) long-chain polyamine synthesis enzymes, which are involved in silica precipitation; (iv) secretion and vesicle trafficking-related proteins, which facilitate the transport of silica precursors to the cell surface; and (v) adhesion and skeletal proteins, which mediate cell-wall assembly and attachment.

**Figure 4:**
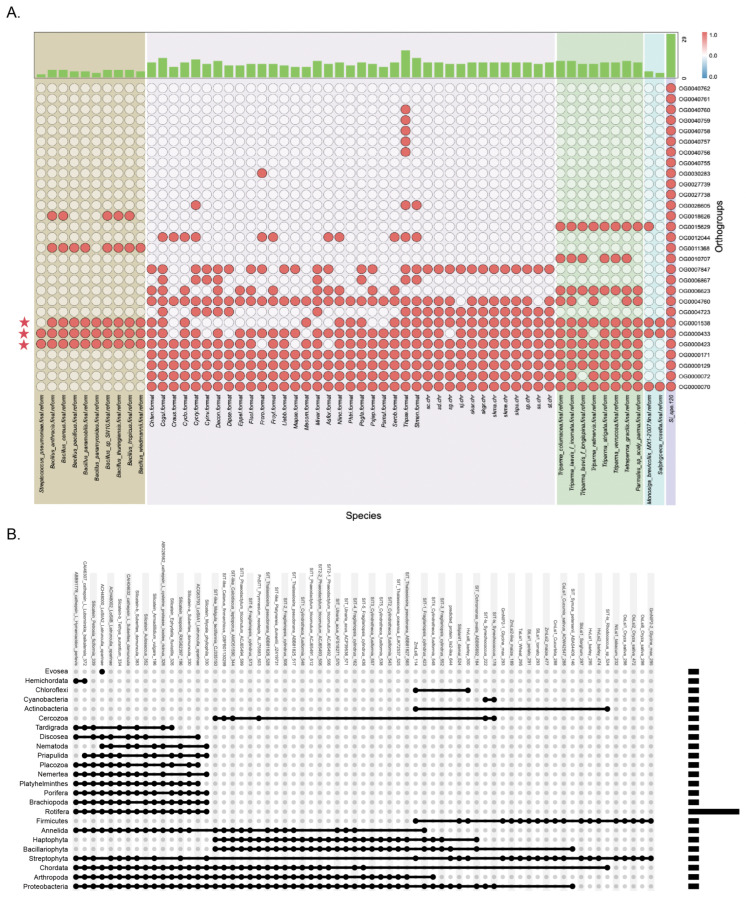
Distribution patterns of known siliceous cell wall-associated genes across four major lineages. (**A**) Bubble plot showing 29 orthogroups containing siliceous cell wall-associated genes across the four silica-bearing lineages. Three orthogroups (star-marked) are shared by *Bacillus*, Bacillariophyta, and choanoflagellates/Parmales. Bubble size indicates the number of species per lineage containing each orthogroup. (**B**) Taxonomic distribution of siliceous cell wall-associated gene homologs from NR database searches (identity > 30%). The heatmap displays presence/absence patterns across major lineages, showing shared distributions between Bacillariophyta and Haptophyta, and between Firmicutes and Streptophyta.

We searched for homologs of these 120 reference sequences across all 57 genomes using DIAMOND BLASTp, and the presence/absence patterns were visualized as a heatmap (Figure 4A). As expected, diatoms (Bacillariophyta) possessed the most extensive repertoire of siliceous cell wall-associated genes. To further explore the phylogenetic distribution of silicon-related genes, we examined the presence of the 120 reference sequences across the four major lineages with documented silica-related traits (Figure 4A). Among the 120 reference sequences, 29 orthogroups were identified. Of these 29 orthogroups, three orthogroups (OG0001538, OG0000433, and OG0000423) were detected across *Bacillus*, Bacillariophyta, and choanoflagellates/Parmales. These encode, respectively: (i) a glycosyltransferase (EXT1, K02366) involved in cell wall glycosylation; (ii) a sulfoquinovosyltransferase (K06119) involved in membrane lipid synthesis; and (iii) a V-type proton ATPase subunit (K02155) involved in vesicular acidification. The remaining 26 orthogroups were largely restricted to Bacillariophyta and choanoflagellates, with most present across the majority of species in these groups. This distribution pattern aligns with the more sophisticated siliceous cell wall formation mechanisms in diatoms and choanoflagellates, which possess fully developed silicon deposition machinery compared to the more limited silica precipitation capabilities of *Bacillus*.

To assess the broader phylogenetic distribution of siliceous cell wall-associated genes, we searched the NCBI NR database with the 120 reference sequences, retaining homologs with sequence identity >30% that had hits in both prokaryotic and eukaryotic lineages. Visualization of the taxonomic origins revealed distinct distribution patterns (Figure 4B). Diatom-derived genes showed consistent homology with sequences from Haptophyta, indicating shared genetic components between these two major phytoplankton lineages. Meanwhile, *Bacillus*-derived silica-associated genes exhibited consistent homology with sequences from Firmicutes and Streptophyta. These patterns suggest that the genetic components involved in silica-related processes have ancient origins, with shared homologs spanning both prokaryotes and eukaryotes across diverse lineages.

### 3.4. Copy Number Variation and Phylogenetic Relationships of Silicon Transporters and Siliceous Cell Wall Structural Proteins

Among the 120 siliceous cell wall-associated genes analyzed, we performed detailed phylogenetic reconstruction for two functionally distinct categories: silicon transporters (SITs, 57 sequences) and structural proteins (32 sequences). These categories were selected because they are the best-annotated and most directly relevant to silicon uptake and cell wall formation (see Section 2.4).

**Figure 5:**
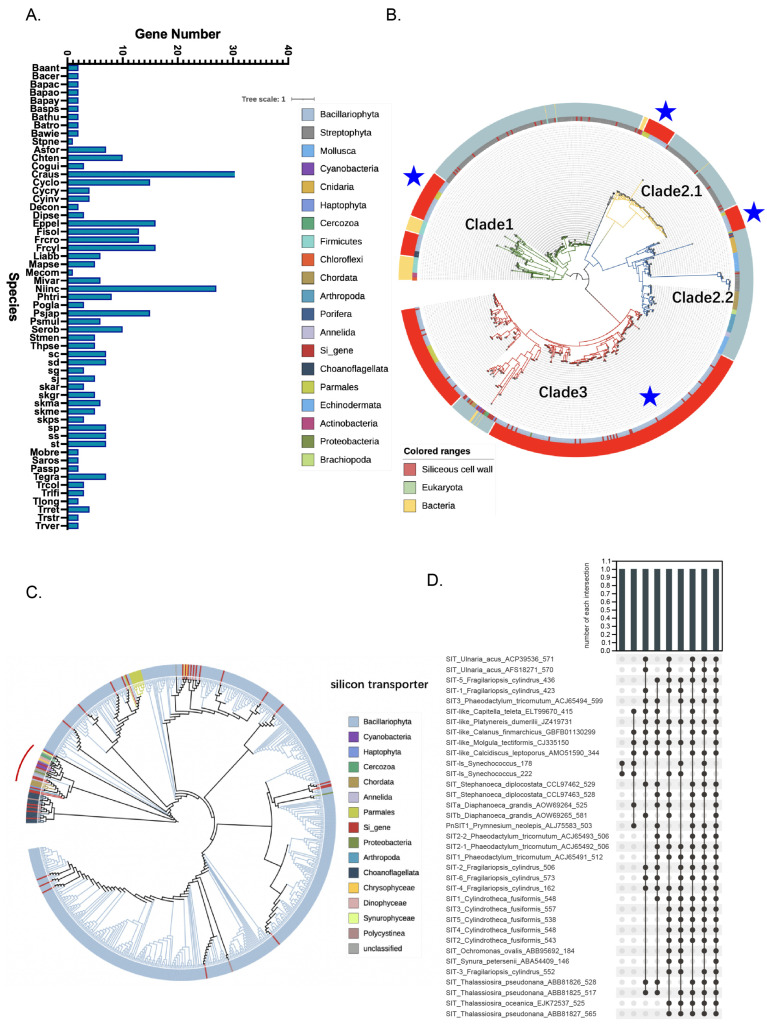
Copy number variation and evolutionary origins of silicon transporters. (**A**) SIT copy number distribution across 57 species. (**B**) Phylogenetic tree of SIT proteins from 57 genomes and NR ffdatabase, showing three major clades (blue stars). (**C**) Expanded tree of Clade 3, highlighting homology between diatom SITs and choanoflagellate/metazoan sequences. (**D**) Taxonomic distribution of Clade 3 SIT homologs from NR database.

Analysis of silicon transporter (SIT) copy numbers across the 57 species revealed marked differences among lineages (Figure 5A). *Bacillus* genomes uniformly harbored only 1 to 2 SIT copies per genome, reflecting the relatively simple silica precipitation capabilities of this bacterial genus. In contrast, Bacillariophyta and Parmales exhibited substantially higher and more variable copy numbers, ranging from as few as 2–3 copies to more than 30 copies per genome. This wide variation suggests lineage-specific expansions of SIT gene families in eukaryotes that have evolved complex siliceous structures, likely driven by the increased demand for silicon uptake and transport during rapid cell wall formation.

Among the 120 siliceous cell wall-associated genes analyzed, 57 were classified as silicon transporters. To investigate the evolutionary history of these transporters, we expanded the dataset by searching for homologs in the NCBI NR database and constructed a maximum likelihood phylogenetic tree (Figure 5B). The resulting tree resolved into three major clades, indicating that SIT proteins have originated multiple times and evolved independently along three distinct evolutionary trajectories. Notably, *Bacillus* SIT sequences formed a distinct clade separate from the eukaryotic sequences, yet showed unexpected phylogenetic affinity with sequences from Streptophyta (land plants and charophyte algae). This topological pattern is consistent with the possibility that *Bacillus* acquired SIT-related genes through horizontal gene transfer (HGT) from streptophyte lineages. For the putative HGT event between *Bacillus* and Streptophyta (SITs), the sequence identity between the *Bacillus* homolog and its closest streptophyte match ranged from 35% to 42% over 60–75% of the query length, consistent with distant homology but not unequivocal evidence for recent transfer. However, alternative explanations—such as ancient paralogy followed by differential loss across lineages, or potential database contamination—cannot be excluded based on phylogenetic evidence alone. The presence of such putative HGT events is consistent with the known propensity of *Bacillus* species to acquire foreign DNA, and may have conferred adaptive advantages in silicon-rich environments.

Further examination of the three major clades revealed distinct distribution patterns (Figure 5C,D). Clade 3, which contained the majority of diatom SIT sequences, showed consistent phylogenetic distribution with the sister lineages of diatoms, with homologs identified across a range of protists and metazoans. Notably, diatom-specific SIT sequences within Clade 3 exhibited close homology to sequences from choanoflagellates—the closest living relatives of animals—as well as from other metazoan lineages. This finding suggests that the SIT gene family in diatoms shares an ancient evolutionary origin with homologs present in the common ancestor of choanoflagellates and animals, rather than having arisen independently within the diatom lineage. The independent evolutionary trajectories of SIT genes across bacteria and eukaryotes, coupled with evidence of horizontal transfer and lineage-specific duplications, support a model in which silicon transporter genes have been acquired and expanded through multiple mechanisms across the tree of life. The presence of conserved SIT homologs in choanoflagellates and metazoans further indicates that the capacity for silicon transport may represent an ancestral eukaryotic trait that has been differentially retained and amplified in lineages that evolved specialized siliceous structures.

**Figure 6:**
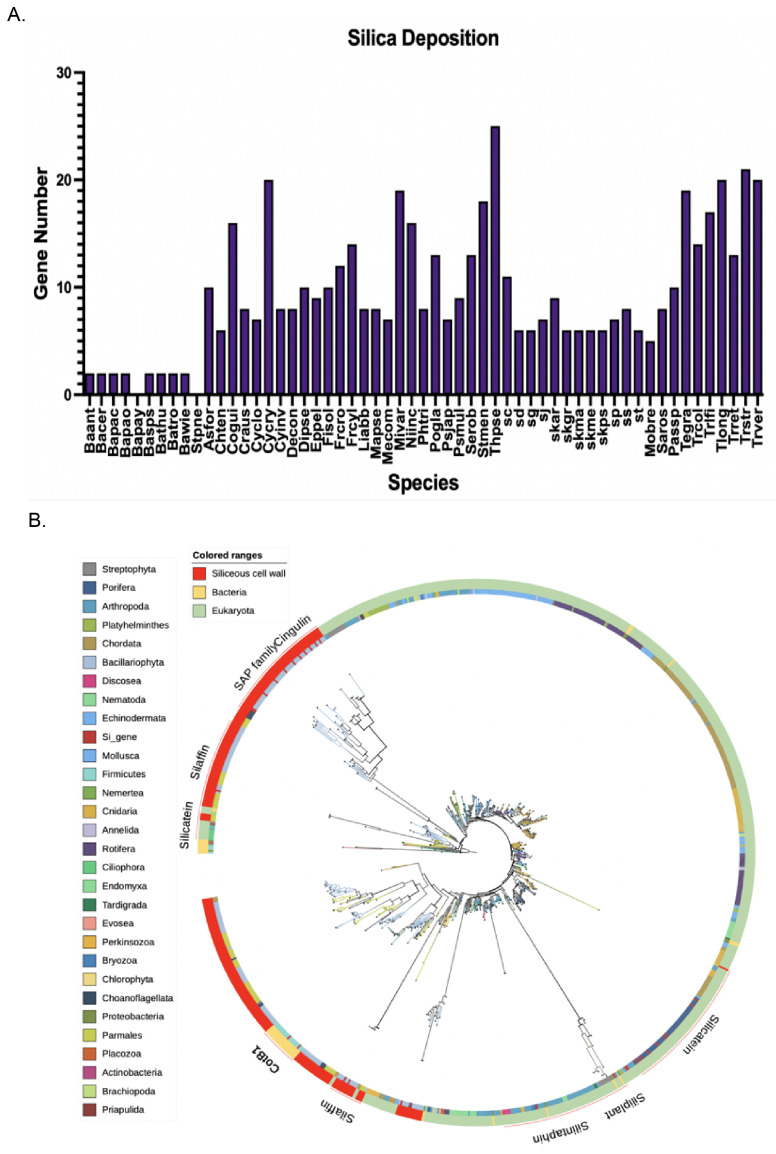
Copy number variation and evolutionary origins of siliceous cell wall structural proteins. (**A**) Copy number distribution of siliceous cell wall structural proteins across 57 species. (**B**) Phylogenetic tree of structural proteins from 57 genomes and the NR database, showing independent putative horizontal gene transfer events from diatoms to *Bacillus* and Proteobacteria.

Analysis of siliceous cell wall structural protein (silica deposition protein) copy numbers across the 57 species revealed a distinct pattern compared to silicon transporters (Figure 6A). *Bacillus* species harbored few or no structural protein homologs, with copy numbers ranging from 0 to 2 per genome, consistent with the limited silica precipitation capabilities of this bacterial genus. In contrast, Bacillariophyta, choanoflagellates, and Parmales exhibited widespread distribution and substantially higher copy numbers, with many species containing multiple structural protein paralogs. This pattern reflects the more sophisticated siliceous cell wall formation mechanisms in these eukaryotic lineages, which require a diverse array of structural components for proper silica deposition and cell wall assembly.

Among the 120 siliceous cell wall-associated genes analyzed, 32 were classified as siliceous cell wall structural proteins. To investigate the evolutionary history of these proteins, we performed phylogenetic analysis including homologs from the NCBI NR database (Figure 6B). The resulting tree revealed a complex evolutionary history, with *Bacillus* sequences clustering separately from the eukaryotic sequences. Notably, *Bacillus* structural protein homologs showed unexpected phylogenetic affinity with sequences from Bacillariophyta, suggesting that *Bacillus* may have acquired these genes through putative horizontal gene transfer (HGT) from diatom lineages [41,42]. Similarly, structural protein homologs from Proteobacteria were found to cluster within diatom-derived clades, indicating a putative HGT event from diatoms to this bacterial phylum. The presence of multiple, phylogenetically distinct HGT events suggests that bacterial acquisition of silica-related genes has occurred repeatedly, potentially conferring adaptive advantages in silicon-rich environments.

Comparison of the phylogenetic trees generated for silicon transporters and siliceous cell wall structural proteins revealed markedly different topological structures. Silicon transporters resolved into three major clades with distinct distribution patterns across bacteria and eukaryotes, while structural proteins exhibited a more complex topology with evidence of multiple independent putative HGT events from diatoms to diverse bacterial lineages. These distinct tree architectures suggest that these two classes of silica-related genes have followed different evolutionary trajectories and were not acquired or expanded through a single, unified mechanism such as endosymbiotic gene transfer [43]. Instead, the independent evolutionary histories of silicon transporters and structural proteins support a model in which the genetic components of siliceous cell wall formation were assembled through multiple independent events, including lineage-specific duplications, horizontal gene transfers, and retention of ancestral genes across deep evolutionary timescales [44,45].

## 4. Discussion

In this study, we constructed a comparative genomic framework across 57 species to investigate the evolutionary origins of siliceous cell wall formation. Our analyses revealed three major findings. First, 75 orthogroups are universally conserved across all sampled species, while 105 orthogroups are consistently present across the four silica-bearing lineages, with functional annotations confirming their roles in fundamental metabolism. Second, among 120 known siliceous cell wall-associated proteins, three orthogroups—encoding a glycosyltransferase (EXT1, K02366), a sulfoquinovosyltransferase (K06119), and a V-type ATPase subunit (K02155)—were broadly distributed across all four silicifying lineages, suggesting ancient evolutionary origins for certain silicon-related components. Third, silicon transporters and structural proteins exhibited markedly distinct evolutionary trajectories, with evidence of lineage-specific duplications and candidate horizontal gene transfers, indicating that the genetic toolkit for silicification was assembled through multiple independent mechanisms.

The phylogenetic reconstructions revealed different evolutionary histories for SITs and structural proteins. SITs resolved into three major clades, with diatom sequences in Clade 3 showing close homology to choanoflagellate and metazoan homologs, suggesting that silicon transport capacity may have been present in the common ancestor of opisthokonts. In contrast, structural proteins showed a more restricted distribution, with highest diversity in diatoms and Parmales, consistent with their specialized roles in silica precipitation. The unexpected clustering of *Bacillus* SIT sequences with Streptophyta, and *Bacillus* structural proteins with diatoms, is consistent with possible HGT events. However, phylogenetic clustering alone does not constitute definitive evidence for HGT. Alternative explanations include ancient paralogy followed by differential loss, convergent evolution of silicon-binding domains, and database contamination. We therefore present these findings as suggestive of possible HGT rather than confirmed transfers.

It is important to distinguish between genes that are directly implicated in silica deposition—such as silicon transporters (SITs), silaffins, and cingulins—and broadly conserved orthogroups that support general cellular functions. The three broadly distributed orthogroups identified in this study (encoding a glycosyltransferase EXT1, a sulfoquinovosyltransferase, and a V-type ATPase subunit) have known roles in cell wall glycosylation, membrane lipid synthesis, and vesicular acidification, respectively. While these functions may be permissive for silicification—for example, vesicular acidification may create an environment conducive to silica transport or precipitation—direct evidence for their active involvement in silica deposition remains lacking. We therefore present these three orthogroups as components of the cellular background that may have been co-opted during the evolution of silicification, rather than as confirmed silicification genes.

Several limitations should be acknowledged. Siliceous sponges were not included due to fragmented genome assemblies. Our sampling of choanoflagellates (*n* = 2) and bacteria (only *Bacillus*) is limited, and conclusions regarding ancient origins should be interpreted with caution. Functional annotations were restricted to Phaeodactylum tricornutum, the best-annotated diatom model. Despite these limitations, our study provides a valuable genomic resource for understanding the evolutionary assembly of siliceous cell wall formation. The finding that certain silicon-related components predate major eukaryotic divergences challenges the view that silicification machinery evolved independently in each lineage. Instead, our data support a model in which ancestral eukaryotes possessed a rudimentary set of silicon-related genes that were subsequently expanded, duplicated, and in some cases horizontally transferred, giving rise to the diverse silicification capabilities observed today.

## 5. Conclusions

In this study, we constructed a comparative genomic framework across 57 species to investigate the evolutionary origins of siliceous cell wall formation. Our findings reveal three major insights. First, 75 core orthogroups are universally conserved across all sampled lineages, while 105 orthogroups are consistently present across the four silica-bearing lineages. Second, three orthogroups—encoding a glycosyltransferase, a sulfoquinovosyltransferase, and a V-type ATPase subunit—are broadly distributed across all four silicifying lineages, suggesting an ancient origin for certain silicon-related components. Third, silicon transporters and structural proteins exhibit distinct evolutionary trajectories, including lineage-specific duplications and candidate horizontal gene transfers, indicating that the siliceous cell wall genetic toolkit was assembled through multiple mechanisms. This study provides a genomic foundation for understanding biomineralization evolution across eukaryotes and offers a valuable resource for future investigations.

## Figures and Tables

**Table 1:** Summary of the Species genome in this study.

Phylum/Class	Species Number
*Bacillus*	10
*Chlorophyta*	4
*Streptophyta*	2
*Rhodophyta*	3
*Alveolata*	3
*Parmales*	8
*Choanoflagellate*	2
*Bacillariophyta*	25

## Data Availability

All data needed to evaluate the conclusions in the paper are present in the paper and/or Supplementary Information. The algal species protein sets are available on the Figshare repository: https://doi.org/10.6084/m9.figshare.28793813 (accessed on 16 April 2025).

## Supplementary Materials

The following supporting information can be downloaded at: https://www.mdpi.com/article/10.3390/biology15141127/s1. Table S1: Summary of genomes included in this study.

## References

[1] Armbrust E.V., Berges J.A., Bowler C., Green B.R., Martinez D., Putnam N.H., Zhou S., Allen A.E., Apt K.E., Bechner M. (2004). The Genome of the Diatom *Thalassiosira pseudonana*: Ecology, Evolution, and Metabolism. Science.

[2] Bowler C., Allen A.E., Badger J.H., Grimwood J., Jabbari K., Kuo A., Maheswari U., Martens C., Maumus F., Otillar R.P. (2008). The Phaeodactylum genome reveals the evolutionary history of diatom genomes. Nature.

[3] Khan S., Scholey J.M. (2018). Assembly, Functions and Evolution of Archaella, Flagella and Cilia. Curr. Biol..

[4] Ševčíková T., Horák A., Klimeš V., Zbránková V., Demir-Hilton E., Sudek S., Jenkins J., Schmutz J., Přibyl P., Fousek J. (2015). Updating algal evolutionary relationships through plastid genome sequencing: Did alveolate plastids emerge through endosymbiosis of an ochrophyte?. Sci. Rep..

[5] Babenko I., Kröger N., Friedrich B.M. (2024). Mechanism of branching morphogenesis inspired by diatom silica formation. Proc. Natl. Acad. Sci. USA.

[6] Blaby-Haas C.E., Merchant S.S. (2019). Comparative and Functional Algal Genomics. Annu. Rev. Plant Biol..

[7] Benoiston A.-S., Ibarbalz F.M., Bittner L., Guidi L., Jahn O., Dutkiewicz S., Bowler C. (2017). The evolution of diatoms and their biogeochemical functions. Philos. Trans. R. Soc. B.

[8] Bachy C., Wittkop P.J., Eggenschwiler J., Groussin M., Beauvais A., Moreira D., López-García P., Maréchal E., Oudart J.B., Boutte C. (2022). The genome of *Thalassiosira pseudonana* reveals extensive horizontal gene transfer. Nat. Commun..

[9] Fan X., Qiu H., Han W., Wang Y., Xu D., Zhang X., Bhattacharya D., Ye N. (2020). Phytoplankton pangenome reveals extensive prokaryotic horizontal gene transfer of diverse functions. Sci. Adv..

[10] Malviya S., Scalco E., Audic S., Vincent F., Veluchamy A., Poulain J., Wincker P., Iudicone D., de Vargas C., Bittner L. (2016). Insights into global diatom distribution and diversity in the world’s ocean. Proc. Natl. Acad. Sci. USA.

[11] Serôdio J. (2021). Diatom motility: Mechanisms, control and adaptive value. Diatom Gliding Motility.

[12] Andersson J.O. (2005). Lateral gene transfer in eukaryotes. Cell. Mol. Life Sci..

[13] Keeling P.J., Palmer J.D. (2008). Horizontal gene transfer in eukaryotic evolution. Nat. Rev. Genet..

[14] Haas B.J., Salzberg S.L., Zhu W., Pertea M., Allen J.E., Orvis J., White O., Buell C.R., Wortman J.R. (2008). Automated eukaryotic gene structure annotation using EVidenceModeler and the Program to Assemble Spliced Alignments. Genome Biol..

[15] Lin S. (2011). Genomics of dinoflagellates. Res. Microbiol..

[16] Stanke M., Keller O., Gunduz I., Hayes A., Waack S., Morgenstern B. (2006). AUGUSTUS: Ab initio prediction of alternative transcripts. Nucleic Acids Res..

[17] Smit A.F.A., Hubley R., Green P. RepeatMasker Open-4.0. 2013–2015. http://www.repeatmasker.org.

[18] Flynn J.M., Hubley R., Goubert C., Rosen J., Clark A.G., Feschotte C., Smit A.F. (2020). RepeatModeler2 for automated genomic discovery of transposable element families. Proc. Natl. Acad. Sci. USA.

[19] Birney E., Clamp M., Durbin R. (2004). GeneWise and Genomewise. Genome Res..

[20] Lukashin A.V., Borodovsky M. (1998). GeneMark.hmm: New solutions for gene finding. Nucleic Acids Res..

[21] Seppey M., Manni M., Zdobnov E.M. (2019). BUSCO: Assessing Genome Assembly and Annotation Completeness. Gene Prediction: Methods and Protocols.

[22] Emms D.M., Kelly S. (2019). OrthoFinder: Phylogenetic orthology inference for comparative genomics. Genome Biol..

[23] Buchfink B., Reuter K., Drost H.-G. (2021). Sensitive protein alignments at tree-of-life scale using DIAMOND. Nat. Methods.

[24] Katoh K., Standley D.M. (2013). MAFFT Multiple Sequence Alignment Software Version 7: Improvements in Performance and Usability. Mol. Biol. Evol..

[25] Nguyen L.-T., Schmidt H.A., Von Haeseler A., Minh B.Q. (2015). IQ-TREE: A Fast and Effective Stochastic Algorithm for Estimating Maximum-Likelihood Phylogenies. Mol. Biol. Evol..

[26] Capella-Gutiérrez S., Silla-Martínez J.M., Gabaldón T. (2009). TrimAl: A tool for automated alignment trimming in large-scale phylogenetic analyses. Bioinformatics.

[27] Letunic I., Bork P. (2019). Interactive Tree of Life (iTOL) v5: An online tool for phylogenetic tree display and annotation. Nucleic Acids Res..

[28] Cantalapiedra C.P., Hernández-Plaza A., Letunic I., Bork P., Huerta-Cepas J. (2021). EggNOG-mapper v2: Functional annotation of large sets of proteins based on evolutionary relationships. Mol. Biol. Evol..

[29] Wu T., Hu E., Xu S., Chen M., Guo P., Dai Z., Feng T., Zhou L., Tang W., Zhan L. (2021). ClusterProfiler 4.0: A universal enrichment tool for interpreting omics data. Innovation.

[30] Keeling P.J. (2013). The Number, Speed, and Impact of Plastid Endosymbioses in Eukaryotic Evolution. Annu. Rev. Plant Biol..

[31] Aranda M., Li Y., Liew Y.J., Baumgarten S., Simakov O., Wilson M.C., Piel J., Ashoor H., Bougouffa S., Bajic V.B. (2016). Genomes of coral dinoflagellate symbionts highlight evolutionary adaptations conducive to a symbiotic lifestyle. Sci. Rep..

[32] Gould S.B., Waller R.F., McFadden G.I. (2008). Plastid Evolution. Annu. Rev. Plant Biol..

[33] Qiu H., Price D.C., Weber A.P., Reeb V., Yang E.C., Lee J.M., Kim S.Y., Yoon H.S., Bhattacharya D. (2013). Adaptation through horizontal gene transfer in the cryptoendolithic red alga *Galdieria phlegrea*. Curr. Biol..

[34] Kominek J., Doering D.T., Opulente D.A., Shen X.-X., Zhou X., DeVirgilio J., Hulfachor A.B., Groenewald M., Mcgee M.A., Karlen S.D. (2019). Eukaryotic Acquisition of a Bacterial Operon. Cell.

[35] Kumar S., Suleski M., Craig J.M., Kasprowicz A.E., Sanderford M., Li M., Stecher G., Hedges S.B. (2022). TimeTree 5: An Expanded Resource for Species Divergence Times. Mol. Biol. Evol..

[36] Evans K.M., Bates S.S., Medlin L.K. (2004). Microsatellite markers for the toxic diatom Pseudo-nitzschia multiseries (Bacillariophyceae). J. Phycol..

[37] Kooistra W.H., Sarno D., Balzano S., Gu H., Andersen R.A., Zingone A. (2008). Global Diversity and Biogeography of *Skeletonema* Species (Bacillariophyta). Protist.

[38] Bussard A., Corre E., Hubas C., Duvernois-Berthet E., Le Corguillé G., Jauffrais T., Jeanthon C., Siano R., Schapira R., Destombe C. (2016). Salinity acclimation in the marine diatom *Phaeodactylum tricornutum*: A physiological and transcriptomic approach. Environ. Microbiol..

[39] Scala S., Carels N., Falciatore A., Chiusano M.L., Bowler C. (2002). Genome Properties of the Diatom *Phaeodactylum tricornutum*. Plant Physiol..

[40] Richards T.A., Dacks J.B., Jenkinson J.M., Thornton C.R., Talbot N.J. (2006). Evolution of Filamentous Plant Pathogens: Gene Exchange across Eukaryotic Kingdoms. Curr. Biol..

[41] Dorrell R.G., Villain A., Perez-Lamarque B., de Kerdrel G.A., McCallum G., Watson A.K., Ait-Mohamed O., Alberti A., Corre E., Frischkorn K.R. (2021). Phylogenomic fingerprinting of tempo and functions of horizontal gene transfer within ochrophytes. Proc. Natl. Acad. Sci. USA.

[42] Raina J.-B., Lambert B.S., Parks D.H., Rinke C., Siboni N., Bramucci A., Ostrowski M., Signal B., Lutz A., Mendis H. (2022). Chemotaxis shapes the microscale organization of the ocean’s microbiome. Nature.

[43] Zhang Y., He M., Pan J. (2025). Axonemal microtubule dynamics in the assembly and disassembly of cilia. Biochem. Soc. Trans..

[44] Yang Z., Zeng X., Zhao Y., Chen R. (2023). AlphaFold2 and its applications in the fields of biology and medicine. Signal Transduct. Target. Ther..

[45] DeLano W.L. (2002). PyMOL: An open-source molecular graphics tool. CCP4 Newsl. Protein Crystallogr..

